# Supplementary material for: Gephyrin filaments represent the molecular basis of inhibitory postsynaptic densities
Source: Nat Commun. 2025 Sep 16;16:8293. doi: 10.1038/s41467-025-63748-w (PMC12441120; doi:10.1038/s41467-025-63748-w)
Supplement: Supplementary file 1 — Supplementary Information [file 41467_2025_63748_MOESM1_ESM.pdf]

Gephyrin filaments represent the molecular basis of inhibitory postsynaptic densities

Arthur Macha[1], Filip Liebsch[1], Emanuel H.W. Bruckisch[1], Nele Burdina[1], Imke von Stülpnagel[1], Konrad Benting[1], Monika Gunkel[1], Elmar Behrmann[1*], Guenter Schwarz[1,2*]

[1]Institute of Biochemistry, Department of Chemistry and Biochemistry, University of Cologne, 50674 Cologne, Germany

[2]Center for Molecular Medicine Cologne (CMMC), University of Cologne, Cologne, Germany

*Correspondence:

Guenter Schwarz, gschwarz@uni-koeln.de

Elmar Behrmann, ebehrman@uni-koeln.de

Table of Content

Supplementary figure 1

Supplementary figure 2

Supplementary figure 3

Supplementary figure 4

Supplementary figure 5

Supplementary figure 6

Supplementary figure 7

Supplementary figure 8

Supplementary figure 9

Supplementary figure 10

Supplementary figure 11

Supplementary figure 12

Supplementary figure 13

Supplementary figure 14

Supplementary figure 15

Supplementary table 1

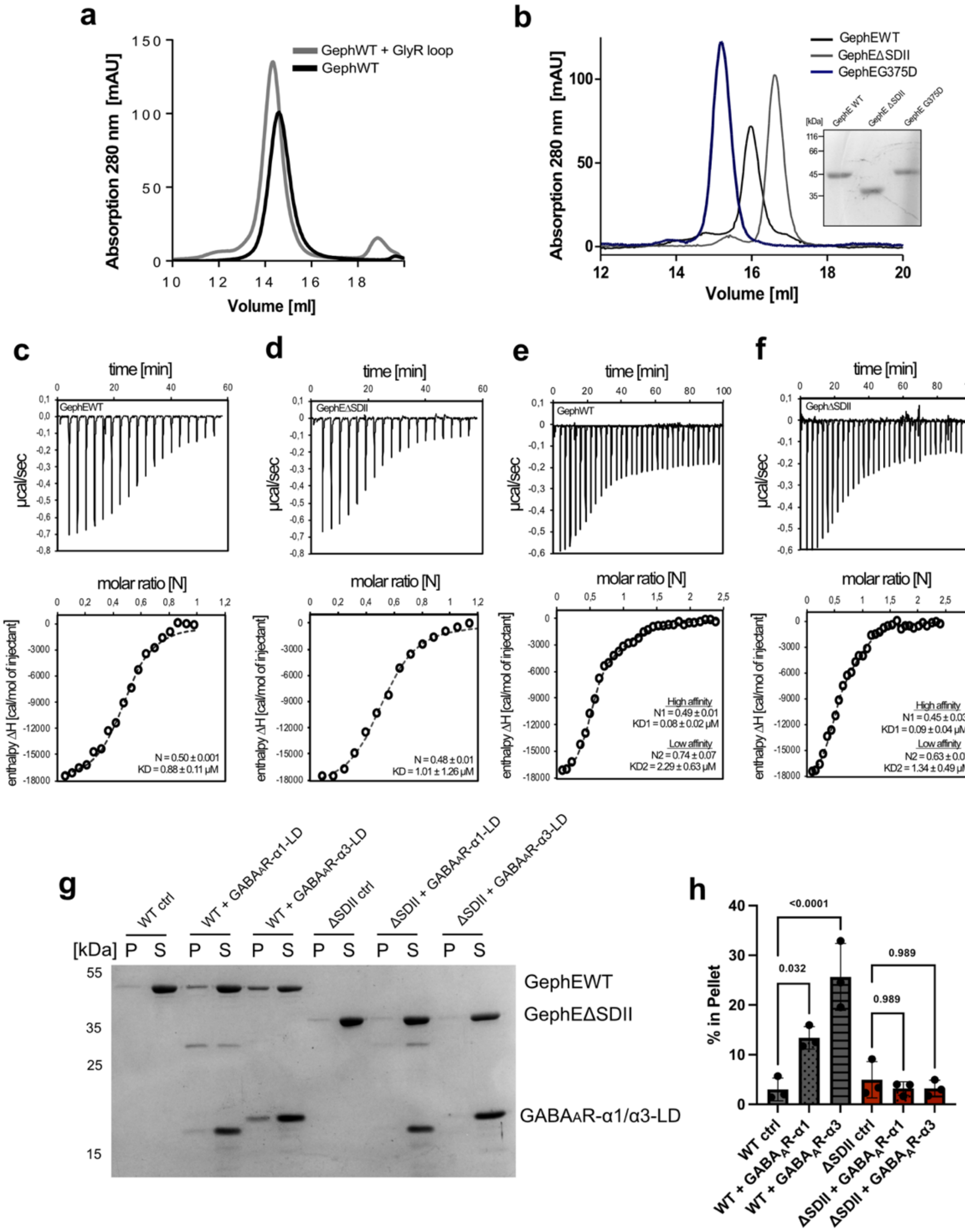

Supplementary Figure 1: Biochemical analysis of gephyrin variants.

a) Representative SEC chromatograms of Geph^{WT} with and without GlyR-loop.

b) Representative SEC chromatograms of GephE^{WT}, GephE^{G375D} and GephE$^{\Delta SDII}$ (added for size comparison). Insert shows SDS-PAGE of respective peak fractions.

c-f) Representative ITC experiments of GephE^{WT} (c), GephE$^{\Delta SDII}$ (d), Geph^{WT} (e), Geph$^{\Delta SDII}$ (f) titrated with GlyR-loop. Raw isotherms are depicted in the upper panel. Respective peak integrations and two-side fit are shown in lower panel with values for binding stoichiometry (N) and dissociation constant (K_D) and respective fitting error following either a one side fit (c, d) or two side fit (e, f) depicting a high and low affinity binding event.

g) Representative SDS-PAGE of sedimentation experiment using GephE and $GABA_AR$ homolog protein ($GABA_AR$-α1-LD, $GABA_AR$-α3-LD) showing the amount of protein present in pellet (P) and supernatant (S) fractions.

h) Quantification of relative GephE amount present in the pellet fraction. Data from three different batches of experiments were presented as means ± SD. Data were analysed by one-way ANOVA ($F_{5,12}$ = 20.34, p = 1.76·10^{-5}), Tukey's post hoc test; n=3, p values of selected comparisons are indicated.

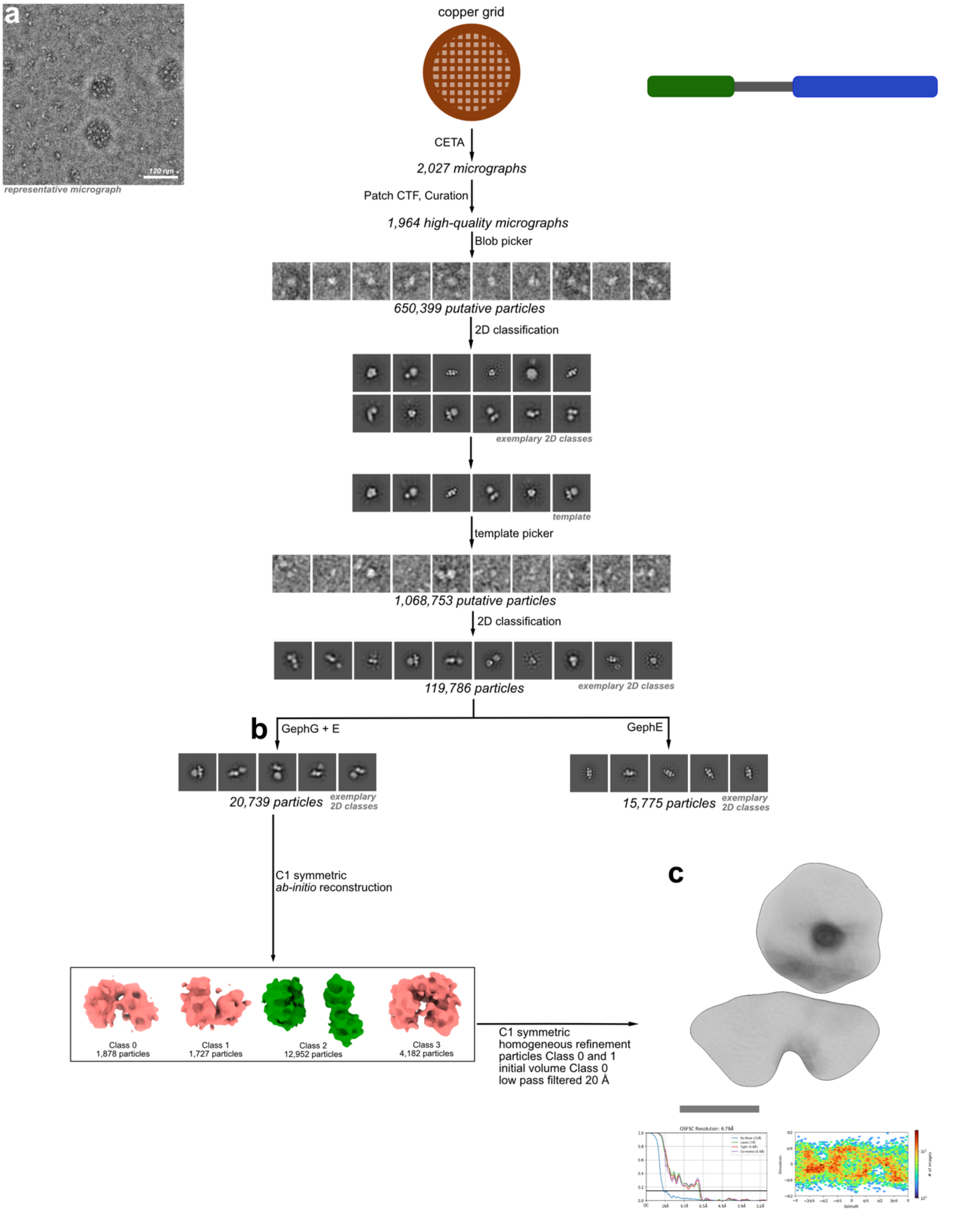

Supplementary Figure 2: Overview of Geph^{WT} negative stain data processing workflow. For the dataset micrographs were picked with good CTF scores and sufficient signal to noise ratio (a). Particles were first picked using a blob picker, and then subjected to unsupervised 2D classification. For 3D reconstruction of gephyrin including both G and E domains (GephG + E) particles from classes showing sharp outlines were used. GephG + E particles were further used for unsupervised ab-initio classification sorting them into four distinct populations (b). From these the particles of one class (shown in green) depicting two distinct densities were subjected to homogenous refinement algorithm with C1 symmetry resulting in a map at approx. 6.8 Å. Shown is the final map low pass filtered to 20 Å (c). Scale bar 50 Å. The cartoon bar diagram illustrates which Geph construct was used in this experiment (green: GephG, grey: GephC, light green: SDII).

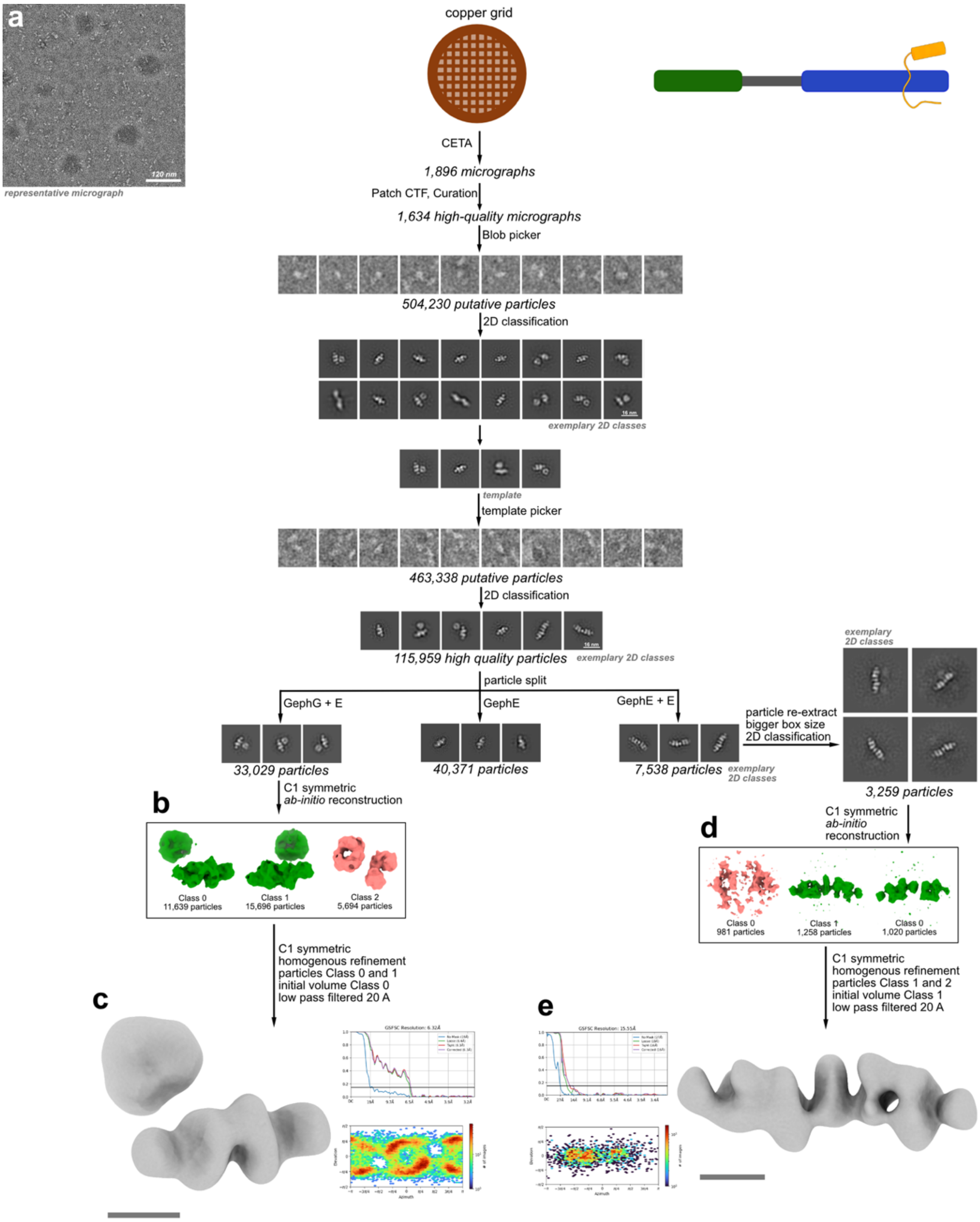

Supplementary Figure 3: Overview of Geph^{WT} in complex with GlyR-loop negative stain data processing workflow. For the dataset micrographs were picked with good CTF scores and sufficient signal to noise ratio (a). Particles were first picked using a blob picker, and then subjected to unsupervised 2D classification continuing with a second supervised 2d classification. For 3D reconstruction of gephyrin including both G and E domains (GephG + E) particles from classes showing sharp outlines were used. GephG + E particles were further used for unsupervised ab-initio classification sorting them into three distinct populations (b). From these the particles of class 0 and 1 (shown in green) depicting two distinct densities were subjected to homogenous refinement algorithm with C1 symmetry resulting in a map at approx. 6.3 Å. Shown is the final map low pass filtered to 20 Å (c). For 3D reconstruction of gephyrin depicting two E domains dimer (GephE + E) particles from classes showing sharp outlines were used. GephE + E particles were further used for unsupervised ab-initio classification sorting them into three distinct populations (d). From these the particles of two classes (shown in green) depicting two distinct densities were subjected to homogenous refinement algorithm with C1 symmetry resulting in a map at approx. 15.6 Å. Shown is the final map low pass filtered to 20 Å (e). Scale bar 50 Å. The cartoon bar diagram illustrates which Geph construct was used in this experiment (green: GephG, grey: GephC, light green: SDII, orange: GlyR-loop).

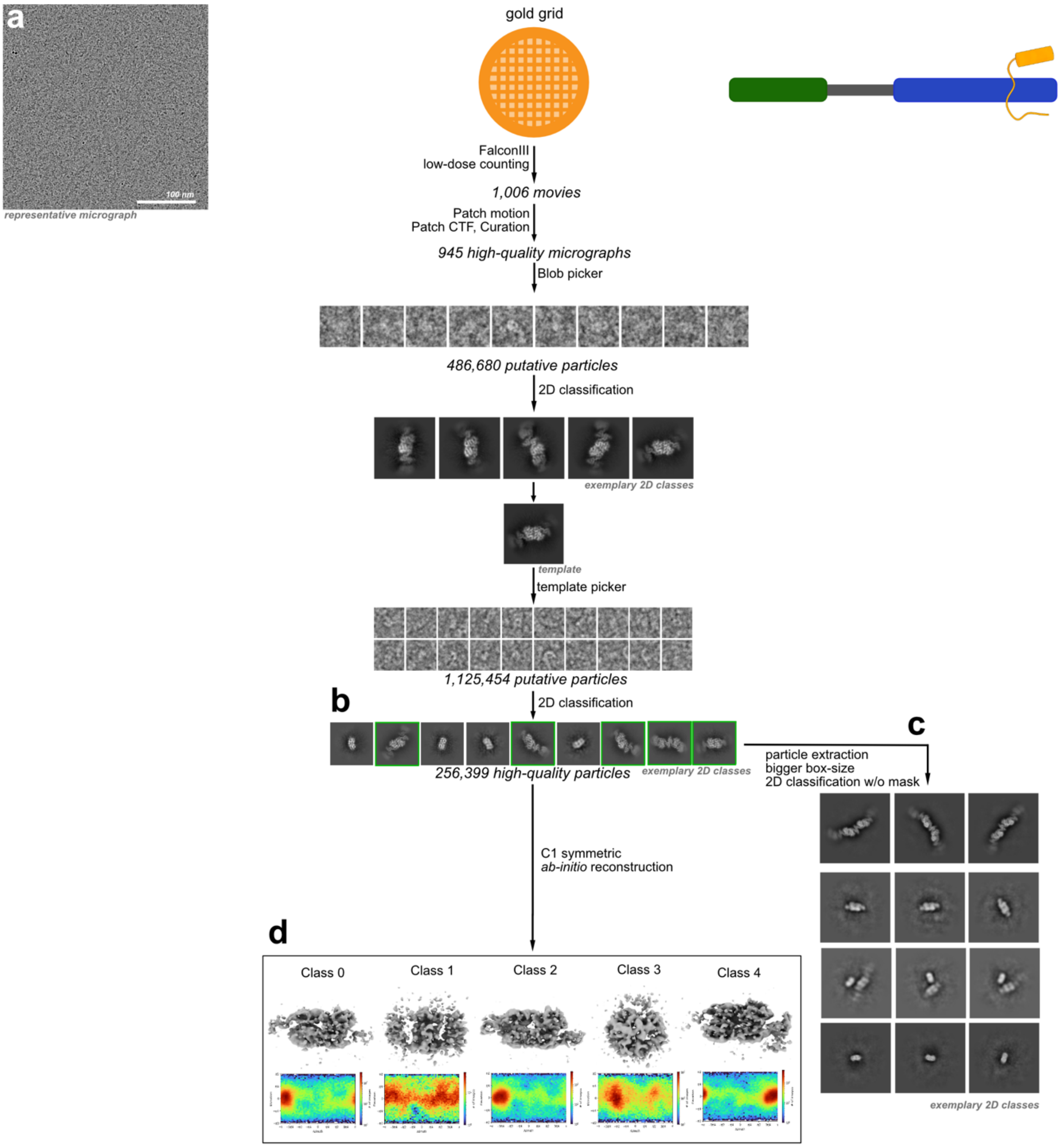

Supplementary Figure 4: Overview of Geph^{WT} in complex with GlyR-loop cryo-EM data processing workflow. For the dataset micrographs were picked with good CTF scores and sufficient signal to noise ratio (a). Particles were first picked using a blob picker, and then subjected to unsupervised 2D classification continuing with a second supervised 2D classification. Resulting 2D classes depicting gephyrin domains either isolated or incorporated into filaments (b). Re-extraction of particles with bigger box-size (512 px) and re-classification depicted the characteristic of gephyrins domains being incorporated into filaments or being isolated on the micrographs (c). Particles from highlighted 2D classes (b, green box) were subjected to unsupervised ab-initio classification sorting them into 5 classes. Reconstruction yielded poor classes with highly fragmented maps and mostly high degree of preferred orientation (d). The cartoon bar diagram illustrates which Geph construct was used in this experiment (green: GephG, grey: GephC, blue: GephE, orange: GlyR-loop).

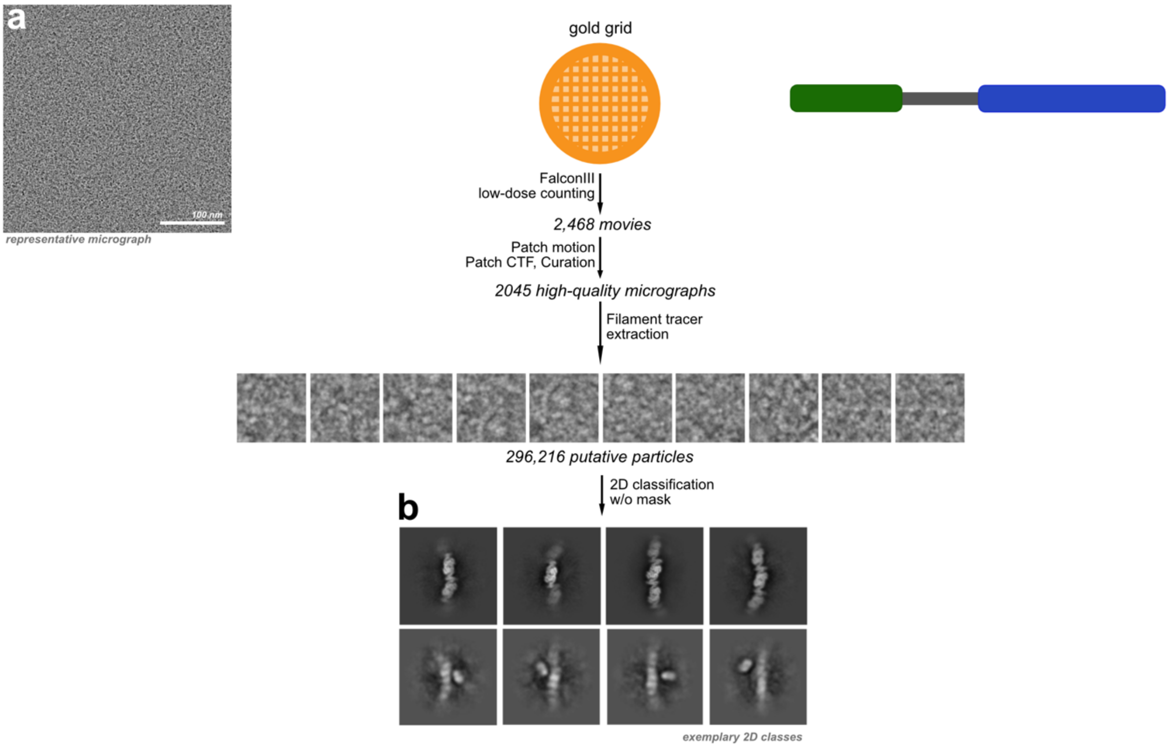

Supplementary Figure 5: Overview of Geph^{WT} without GlyR-loop cryo-EM data processing workflow. For the dataset micrographs were picked with good CTF scores and sufficient signal to noise ratio (a). Particles were first picked using a blob picker and extracted using 512 px box-size and then subjected to unsupervised 2D classification. The resulting 2D class averages depicting gephyrin molecules being incorporated into filaments (b). The cartoon bar diagram illustrates which Geph construct was used in this experiment (green: GephG, grey: GephC, blue: GephE).

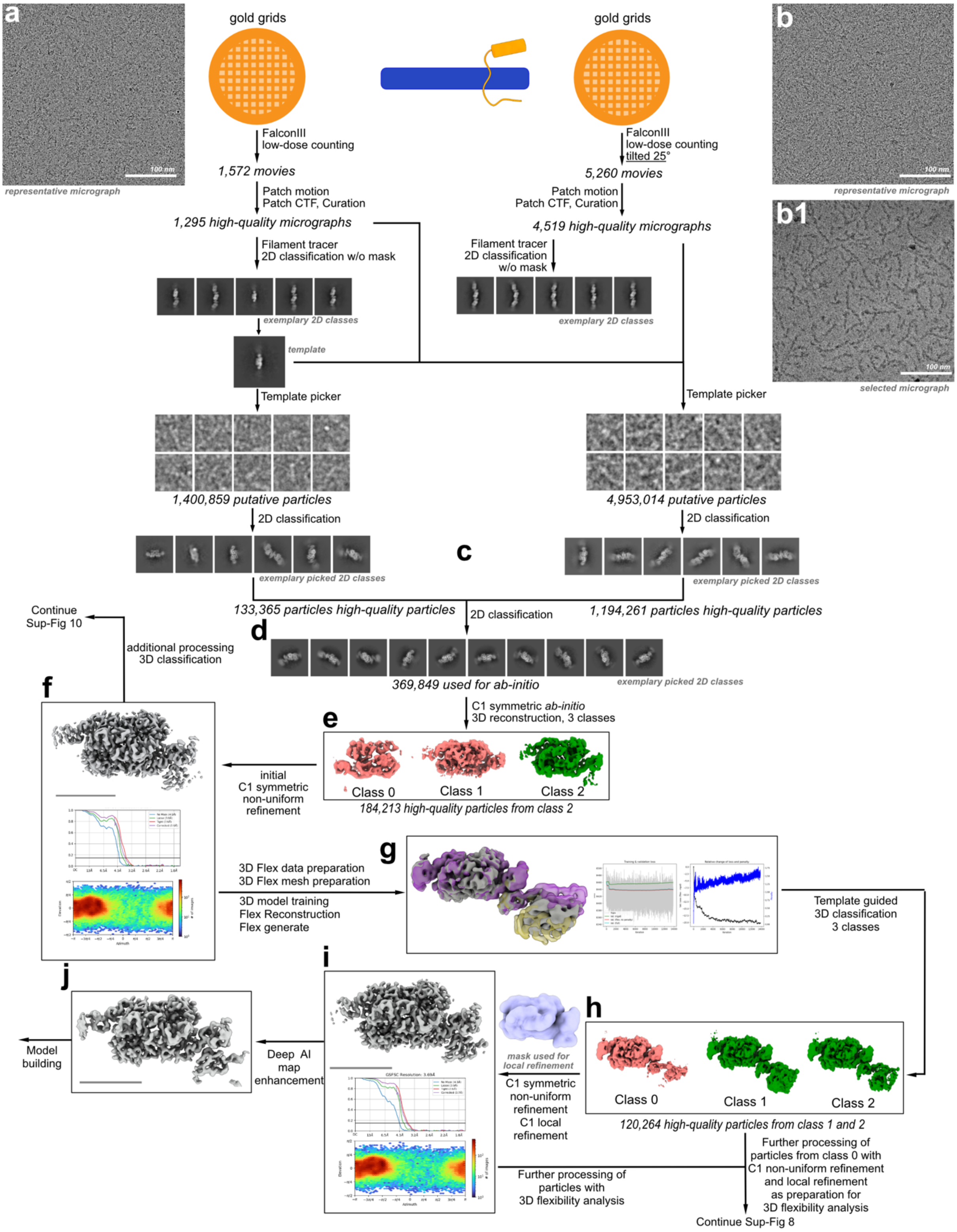

Supplementary Figure 6: Overview of GephE^{WT} in complex with GlyR-loop cryo-EM data processing workflow. For analysis an un-tilted dataset was combined with a dataset recorded at 25° stage tilt to increase the orientations of particles on the micrographs. Subsequently, micrographs were picked with good CTF scores and sufficient signal to noise ratio (a, b). While crowding prevented the clear identification of filaments on most micrographs, several showed clearly visible filaments (b1). Both datasets yielded 2D class averages of good quality depicting GephE incorporated into filaments (c). In a final 2D classification step high-quality particles from both datasets were combined and high-quality particles were picked for ab-initio classification (d). Particles were classified into four classes using unsupervised ab-initio classification of which one class showed coherent density map which included the filament interface (e). Particles from this class were subjected to unsupervised non-uniform refinement which resulted in a map with a global resolution of 3.6 Å (f). The Map and particles were

further used for 3D flexibility reconstruction which yielded density maps showing the SDII interface and adjacent GephE dimer in various positions (g). Two most excessive positions (yellow, purple) and a position occupying a position in between (grey) were chosen for template guided 3D classification of particles used in initial non-uniform refinement (h). 3D classification showed two classes with strong density depicting the filament interface and adjacent GephE dimer and one with weak density (h). Particles from two classes with strong density for the filament interface were further used for non-uniform refinement and subsequent local-refinement utilizing a mask covering the core part of one GephE dimer and the SDII-SDII` interface (i). The resulting map showed high resolution in the core part of the protein and coherent density at the SDII-SDII` interface with a global resolution of 3.7 Å (i). As a final step the resulting map was subjected to deep AI map enhancement for map sharpening which was finally used for atomic model building (j). Scale bars 50 Å. The cartoon bar diagram illustrates which Geph construct was used in this experiment (blue: GephE, orange: GlyR-loop).

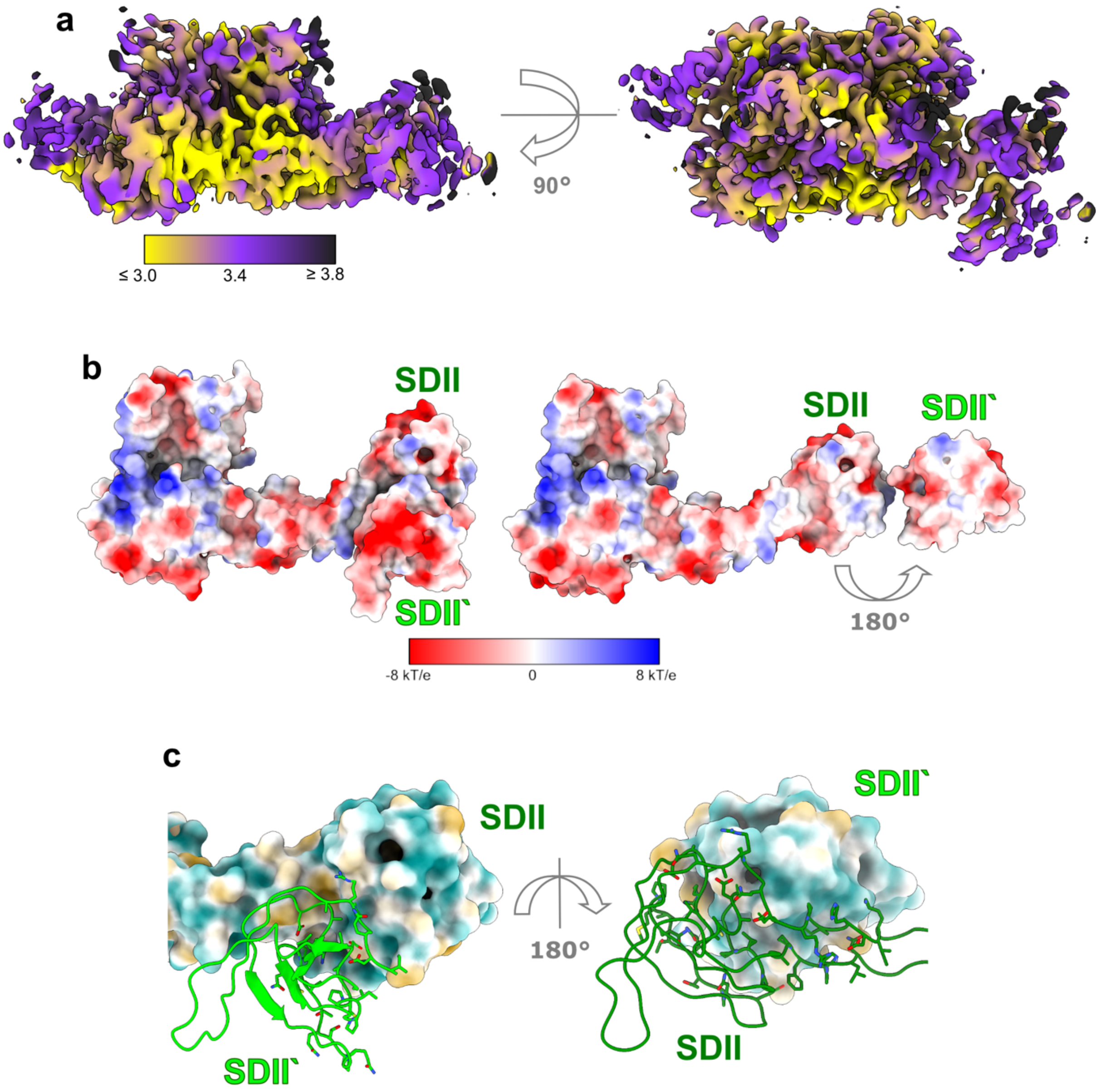

Supplementary Figure 7: Characterization of gephyrin filament interface.
a) Local resolution of final map of GephE dimer (reconstruction from Sup-Figure 6) incorporated into filaments. Colour code indicates resolution [Å].
b) Electrostatic potential distribution of one GephE monomer with filament interface in surface presentation. For better visualization of electrostatic potential of the filament interface, SDII` was turned 180° (right penal). Colour code indicates electrostatic potential [kT/e].
c) Hydrophobicity distribution of GephE interface SDII in surface presentation with SDII` in cartoon presentation (light green, left side) and SDII` in surface presentation with SDII in cartoon presentation (dark green; right side).

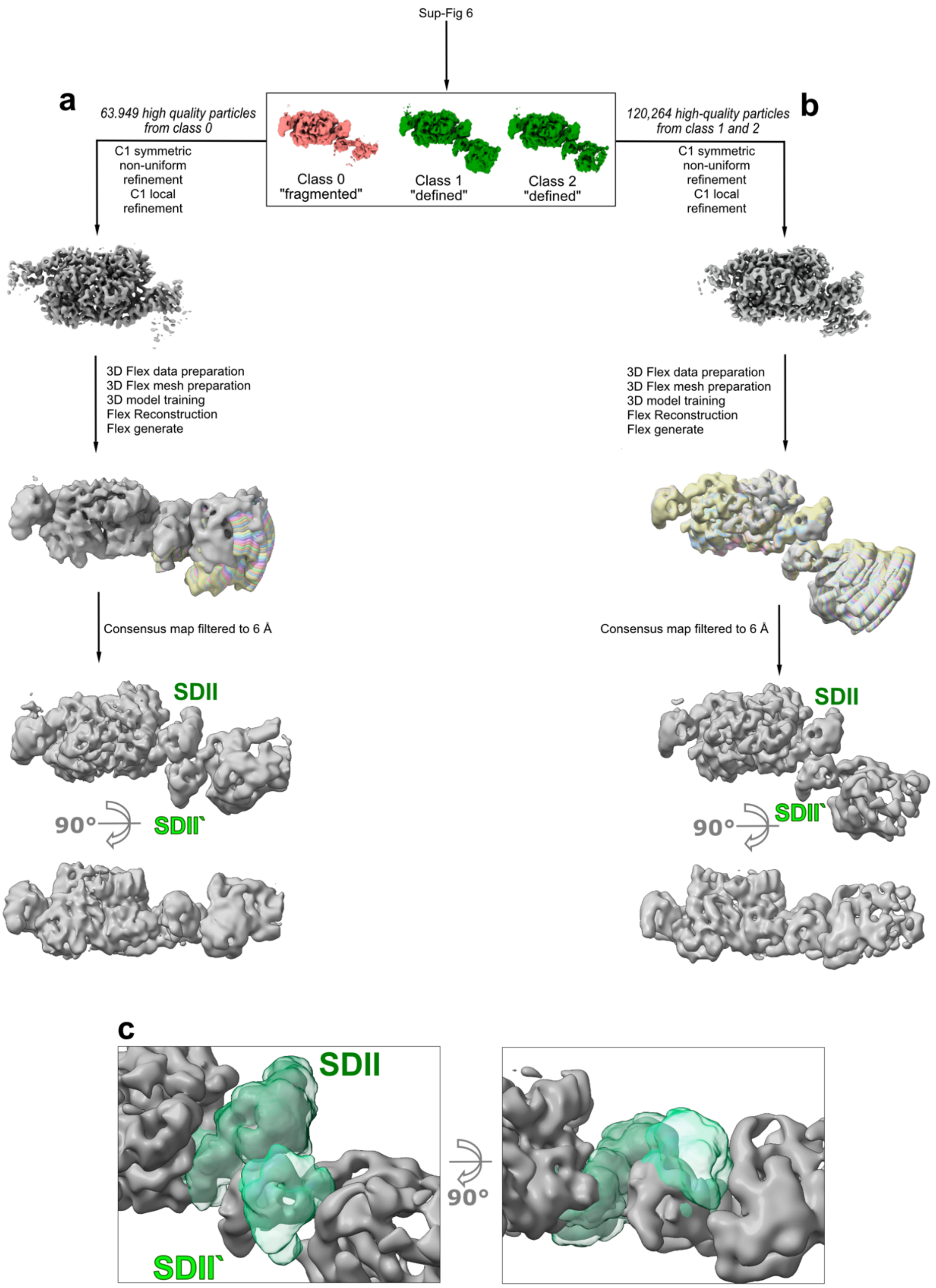

Supplementary Figure 8: Workflow motion-based deep generative modelling of GephE incorporated into filaments.

a, b) Analysis workflow of particles from ab-initio classification depicting either weak (a) or strong (b) density in GephE filament interface region. Colours of overlaying density maps indicating different interface conformations.

c) Alignment of consensus maps depicting the positions of SDII and SDII`. Consensus map from b is shown in surface representation (grey) with overlaying positions of SDII and SDII` from a (green).

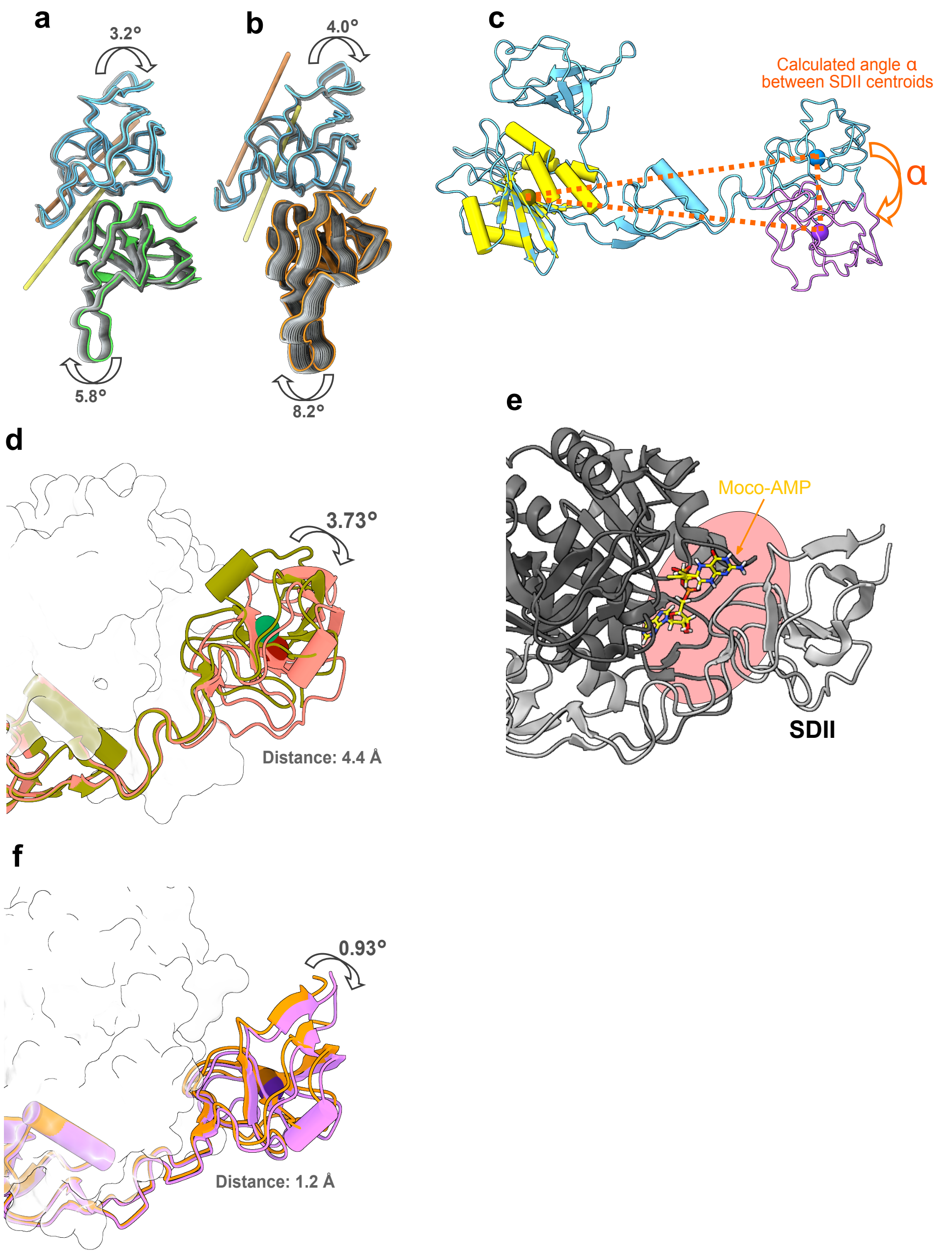

Supplementary Figure 9: Analysis of SDII flexibility.

a, b) Visualization of secondary trajectories depicting possible degrees of freedom of SDII (blue) and SDII` (green or orange) in the main (a) and alternative (b) binding positions based on motion-based deep generative modelling. Strongest deviation from the consensus position is highlighted in colour, the course of movement shown in shades of grey, and the difference between the extreme positions indicated in degree.
c) Visualization of the process by which the positional shift between the SDIIs from different models was measured. Respective atomic structures were aligned to the secondary structure elements of SDIII (selection marked in yellow). The distances between the centroids of SDIII (yellow), SDII (blue) and SDII in the shifted position (purple) were then measured, and by applying trigonometry the angle α was calculated as a proxy for the positional shift between the SDIIs. Note that in this panel for better visualization SDI, SDIII and SDIV from the GephE monomer of shifted position (purple) were removed and change in SDII position was overexaggerated.
d) Alignment of GephE with bound GlyR-loop (rosa, PDB 2FTS) and GephE without GlyR-loop (olive, PDB 2FU3). Changes in angular position and distance between SDII centroids are indicated.
e) Depiction of Moco-AMP (stick representation, yellow) in the active centre of the GephE dimer. GephE monomers are shown different shades of grey. The pink region highlights the active centre relevant for Moco biosynthesis.
f) Alignment of Cnx1E with bound Moco-AMP (purple, PDB 6Q32) and Cnx1E without Moco-AMP (orange, PDB 5G2R). Changes in angular position and distance between SDII centroids are indicated.

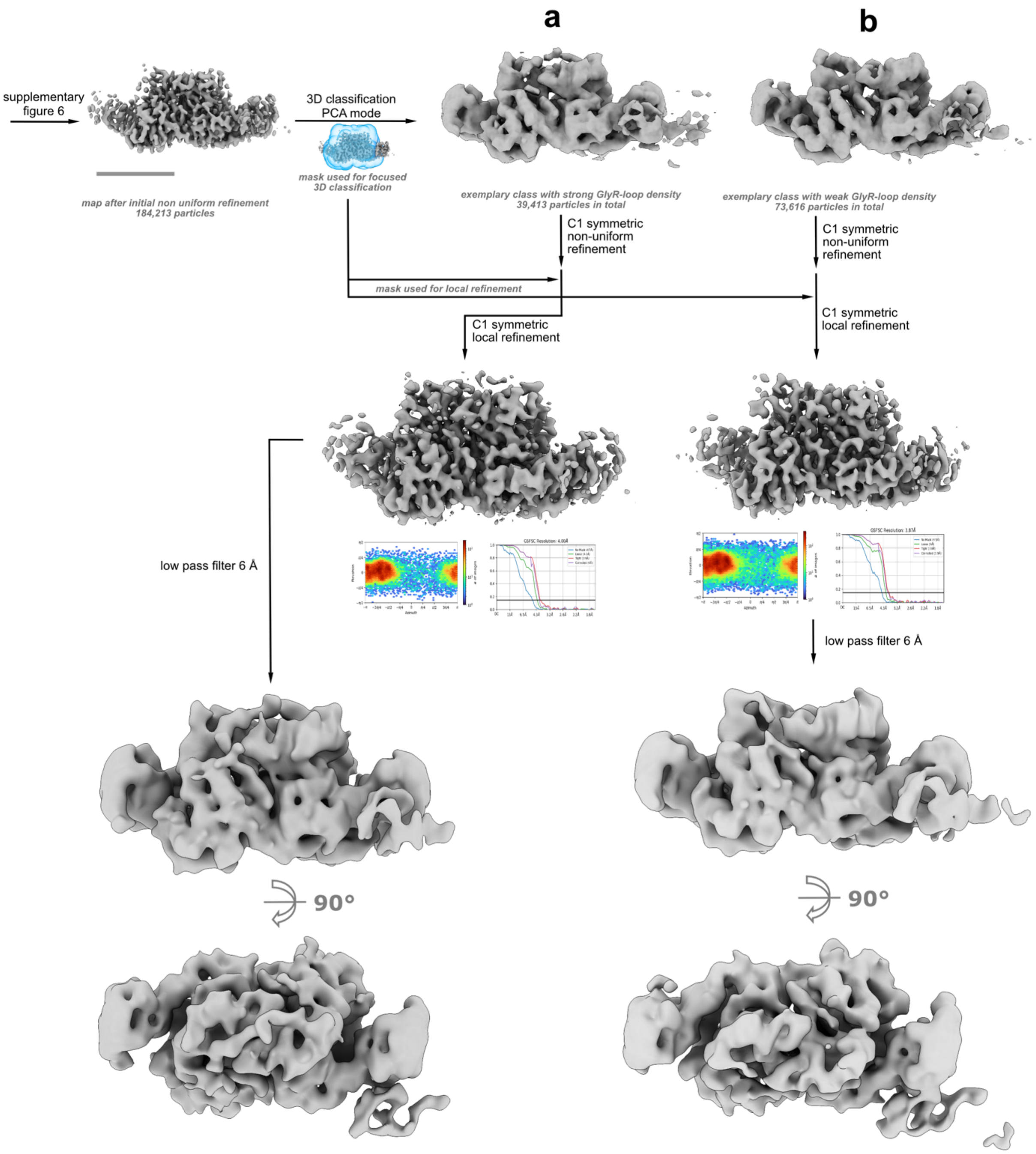

Supplementary Figure 10: Workflow of particle processing to elucidate position of GlyR-loop bound to GephE dimer. Particles from initial reconstruction which were used for initial 3D reconstruction (see Sup-Figure 6) were subjected to 3D classification utilizing a mask focussing on the core part of GephE dimer. Particles from classes showing strong density for GlyR-loop (a) were separately processed from particles with weak or no density for GlyR-loop (b). Both sets of particles were subjected to non-uniform and local refinements. Local refinements were performed with mask focussing on core part of the dimer. The resulting maps were low pass filtered to 6 Å. Scale bar 50 Å.

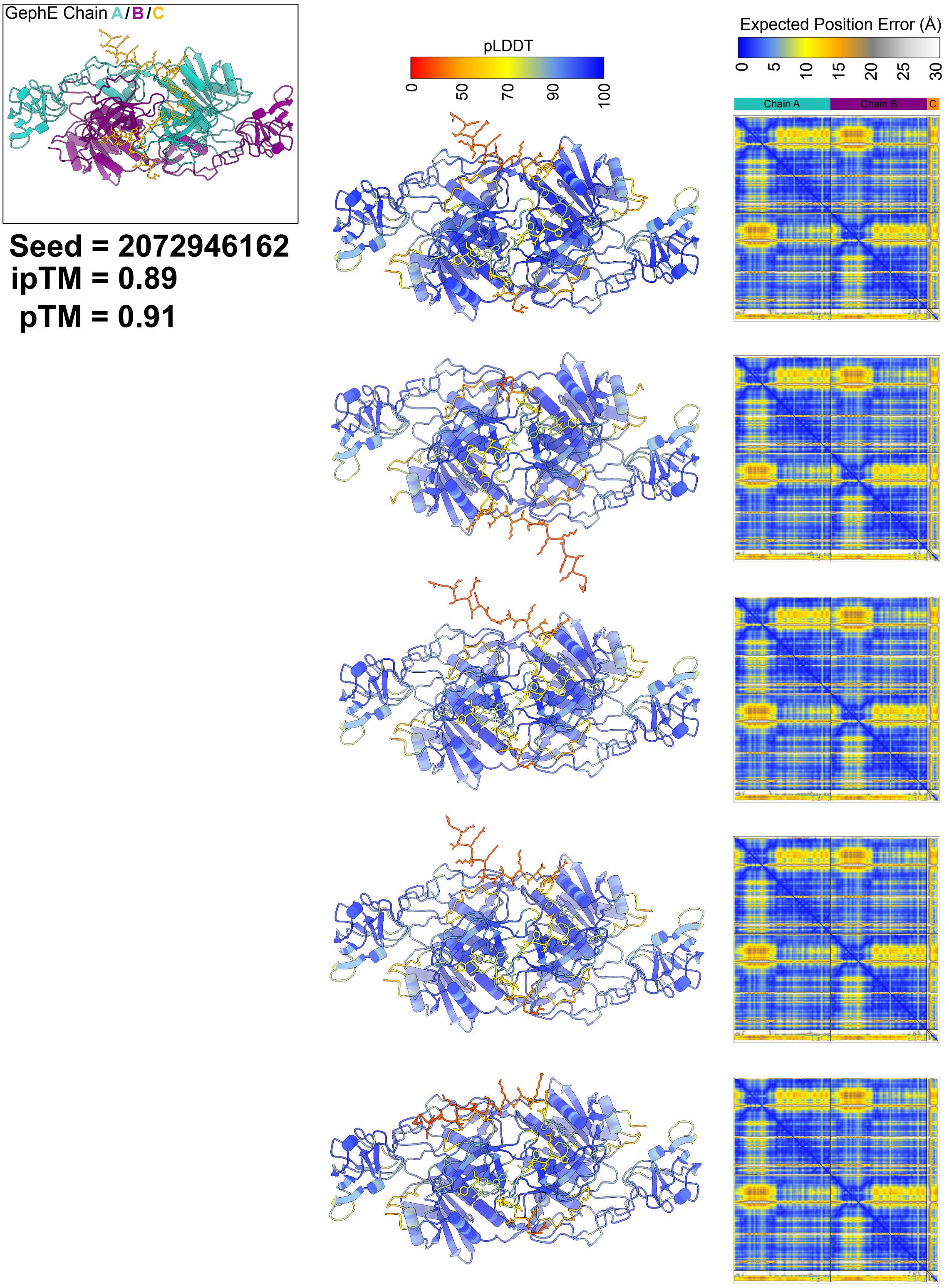

Supplementary Figure 11: Overview of Alphfold 3 quality scores of GephE dimer in complex with GlyR-loop. Shown are the top five prediction models with predicted aligned error (PAE) and predicted local distance difference test (pLDDT). All structures were aligned as shown in the insert. GephE dimer (Chain A, B) and GlyR-loop (Chain C) are shown in cartoon and in stick representations respectively.

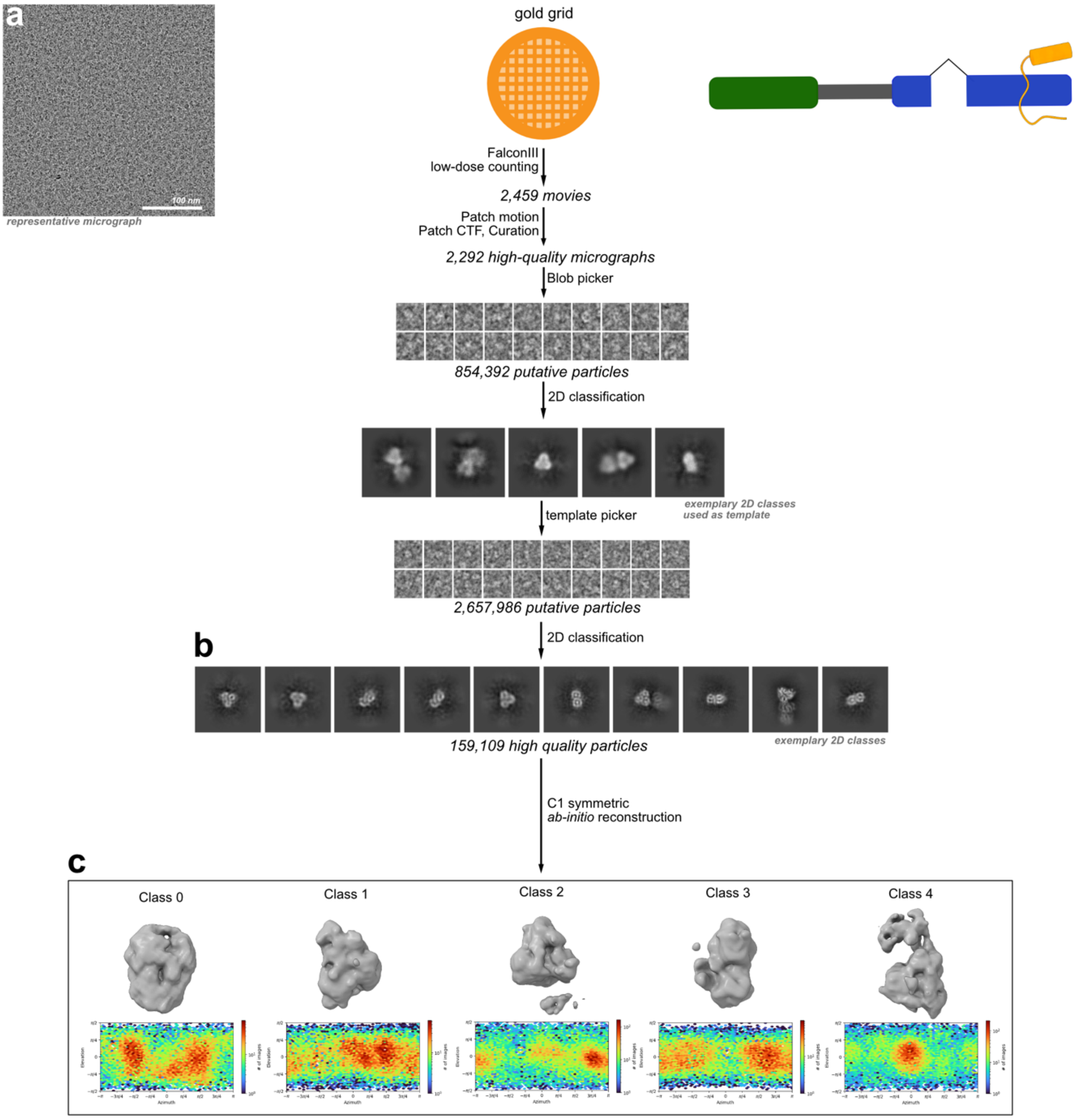

Supplementary Figure 12: Overview of Geph$^{\Delta SDII}$ with GlyR-loop cryo-EM data processing workflow. For the dataset micrographs were picked with good CTF scores and sufficient signal to noise ratio (a). Particles were first picked using a blob picker and subjected to unsupervised 2D classification followed by a second iteration of supervised 2D classification. The resulting 2D class averages depicting isolated particles from various angles along the rotational long axis of the protein (b). High quality particles were used for unsupervised ab-initio reconstruction classifying them into 5 classes. None of the resulting ab-initio classes depicted a map of high quality which could be further processed to high resolution. The cartoon bar diagram illustrates which Geph construct was used in this experiment (green: GephG, grey: GephC, blue: GephE, orange: GlyR-loop).

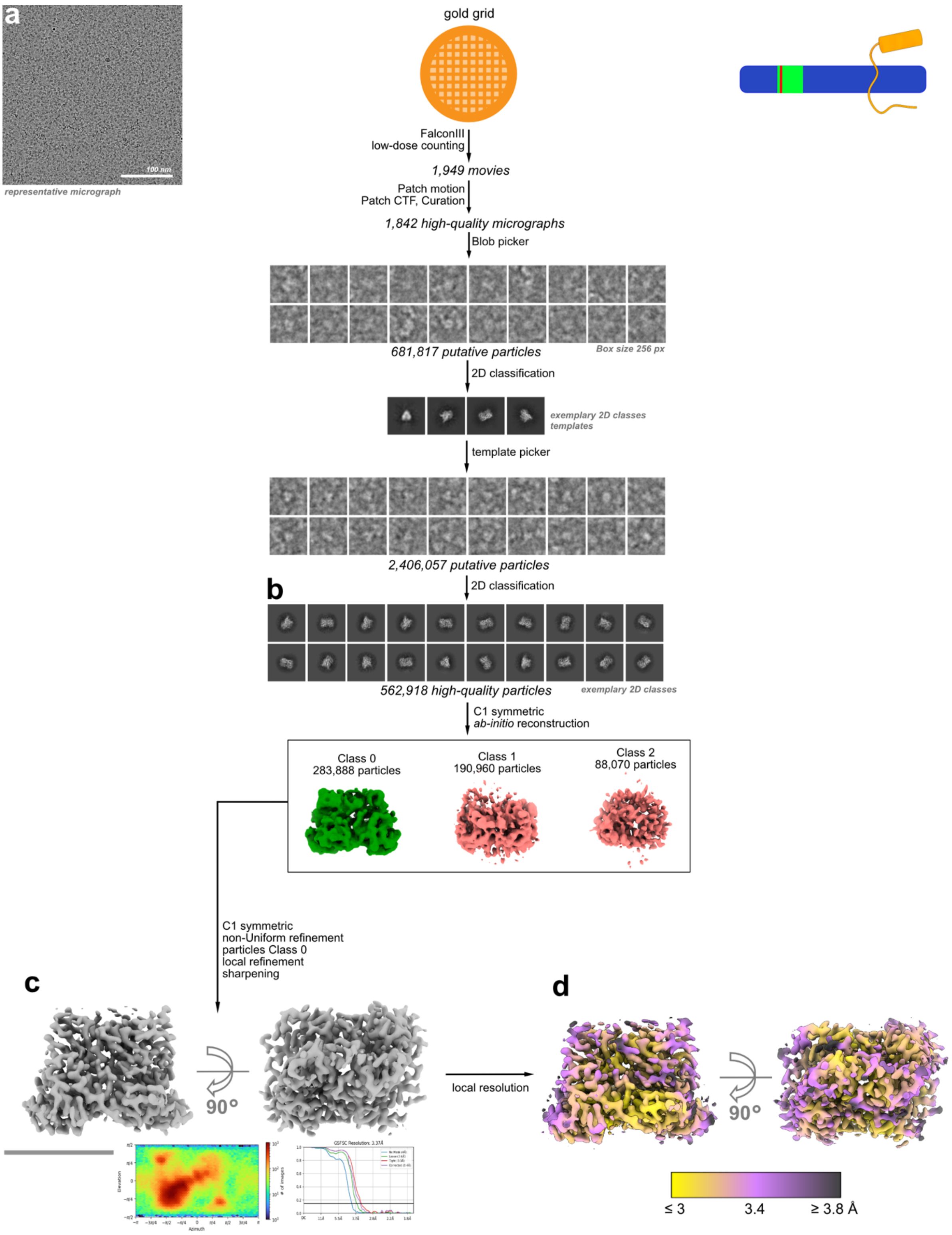

Supplementary Figure 13: Overview of GephE^{G375D} with GlyR-loop cryo-EM data processing workflow. For the dataset micrographs were picked with good CTF scores and sufficient signal to noise ratio (a). Particles were first picked using a blob picker and subjected to unsupervised 2D classification. The resulting 2D class averages depicting isolated particles from various angles (b). High quality particles were used for unsupervised ab-initio reconstruction classifying them into three classes. Particles from best ab-initio class, showing cohesive map and sufficient particle orientations, were further used for non-uniform and local refinement (c). Final map had a local resolution of approx. 3 – 3.8 Å (d). The cartoon bar diagram illustrates which Geph construct was used in this experiment (blue: GephE, light green: SDII, red: position of amino acid substitution, orange: GlyR-loop).

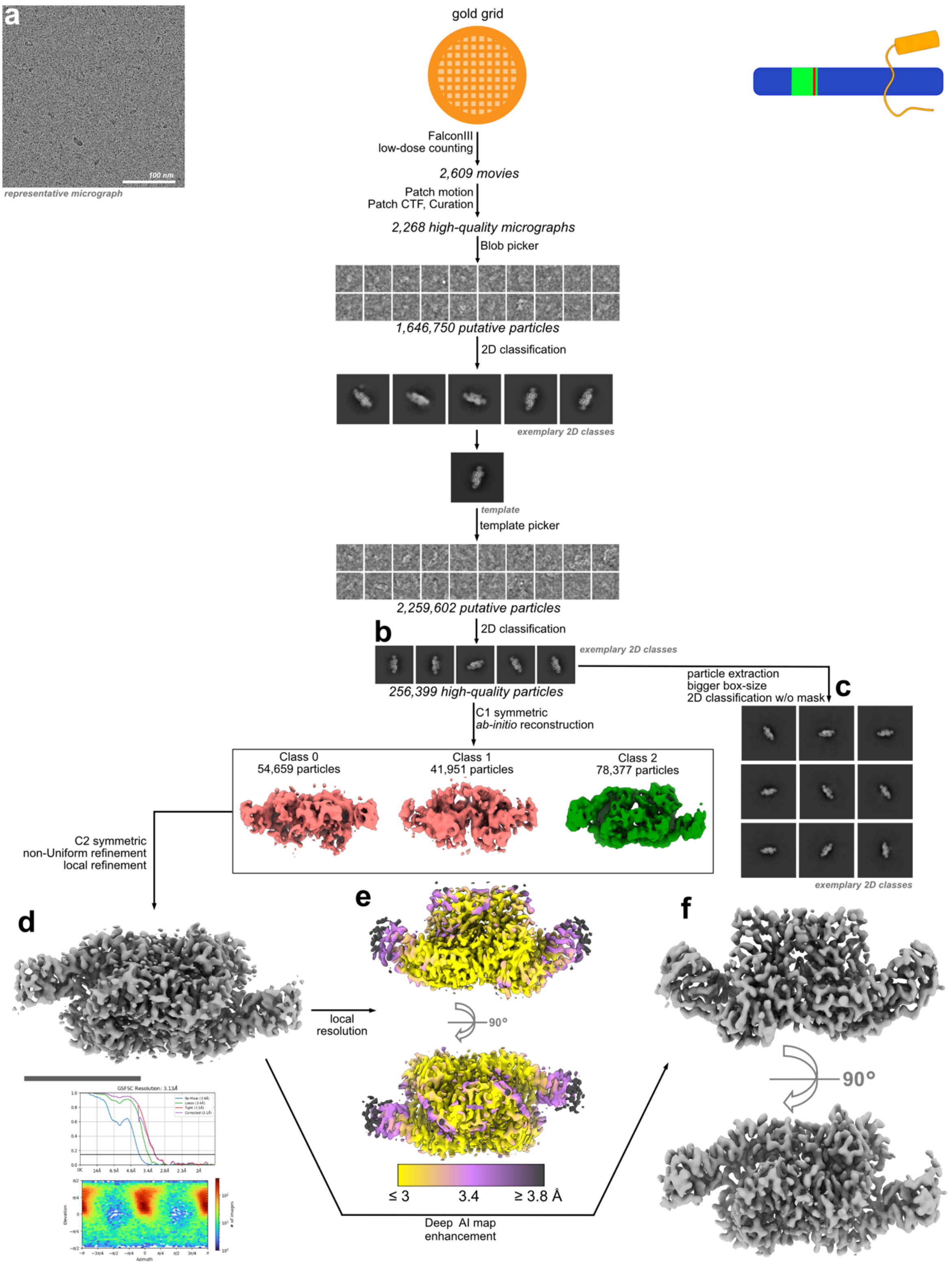

Supplementary Figure 14: Overview of GephE^{D422N} with GlyR-loop cryo-EM data processing workflow. For the dataset micrographs were picked with good CTF scores and sufficient signal to noise ratio (a). Particles were first picked using a blob picker and subjected to unsupervised 2D classification followed by a second iteration of supervised 2D classification. The resulting 2D class averages depicting isolated particles from various angles along the rotational long axis of the protein (b). For comparison high quality particles were re-extracted with bigger box-size (512 px) and subjected to 2D classification. Resulting 2D class averages depicted highly isolated particles (c). High quality particles were used for unsupervised ab-initio reconstruction classifying them into three classes. Particles from best ab-initio class, showing cohesive map, were further used for non-uniform and local refinements with applied C2 symmetry (d). Final map had a local resolution of approx. 3 – 3.8 Å (e). For further map sharpening final map was subjected to deep AI map enhancement to adjust for minor anisotropy in the map (f). The cartoon bar diagram illustrates which Geph construct was used in this experiment (blue: GephE, light green: SDII, red: position of amino acid substitution, orange: GlyR-loop).

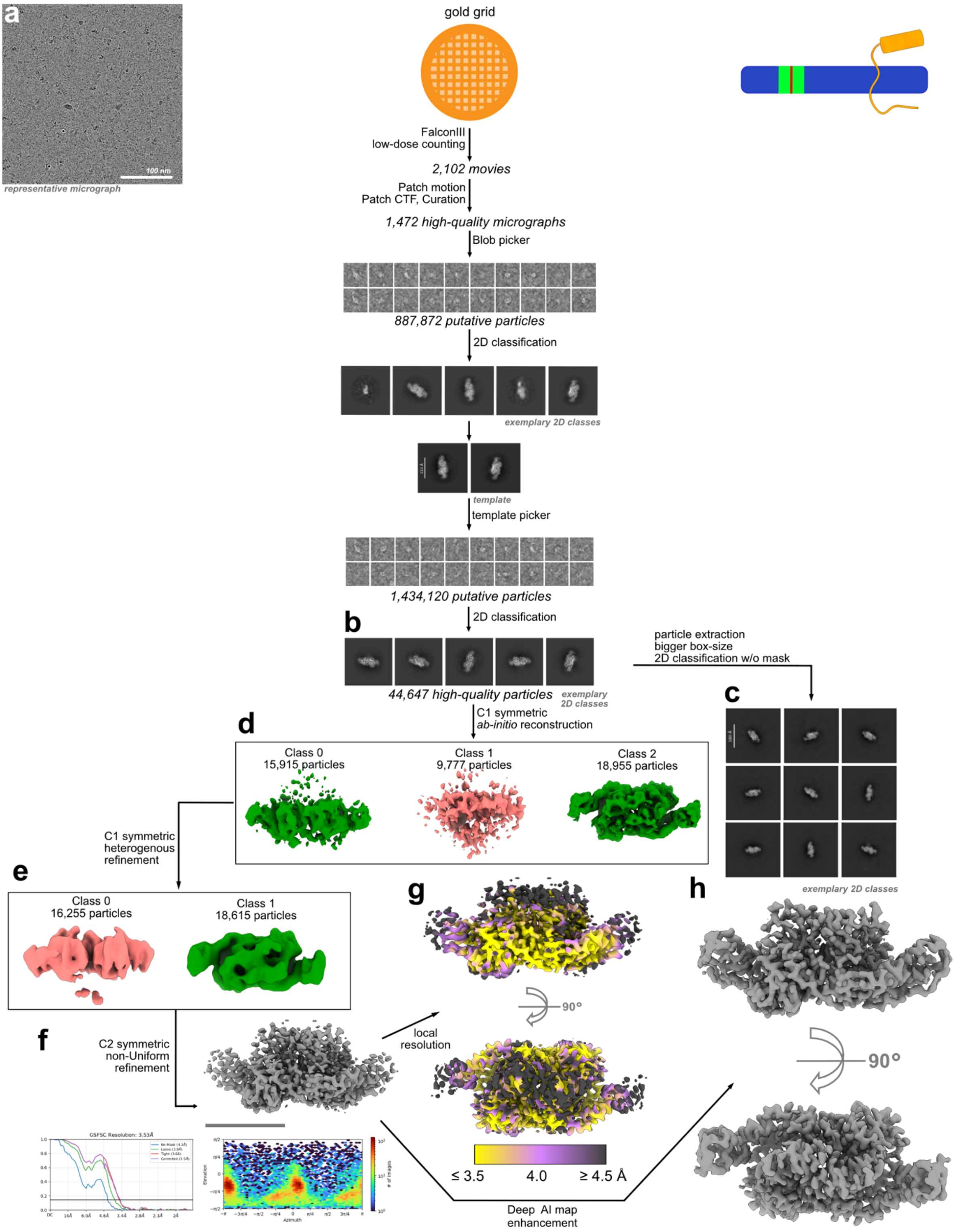

Supplementary Figure 15: Overview of GephE^{R379D} with GlyR-loop cryo-EM data processing workflow. For the dataset micrographs were picked with good CTF scores and sufficient signal to noise ratio (a). Particles were first picked using a blob picker and subjected to unsupervised 2D classification followed by a second iteration of supervised 2D classification. The resulting 2D class averages depicting isolated particles from various angles along the rotational long axis of the protein (b). For comparison high quality particles were re-extracted with bigger box-size (512 px) and subjected to 2D classification. Resulting 2D class averages depicted highly isolated particles (c). High quality particles were used for unsupervised ab-initio reconstruction classifying them into three classes (d). Particles from best ab-initio class, showing cohesive map, were further used for heterogonous refinement with two classes (e). Particles from heterogenous refinement class 1 were used for further non-uniform refinement with applied C2 symmetry (f). Final map had a local resolution of approx. 3.5 – 4.5 Å (g). For further map sharpening final map was subjected to deep AI map enhancement to adjust for minor anisotropy in the map (h). Scale bar 50 Å. The cartoon bar diagram illustrates which Geph construct was used in this experiment (blue: GephE, light green: SDII, red: position of amino acid substitution, orange: GlyR-loop).

Supplementary Table 1: Statistics and additional information of the refinement and model building process

GephE^{WT}-GlyR loop	untilted	25° tilt
**Sample conditions**		
Sample concentration	0.5 mg/ml	
GlyR-loop	3x fold excess	
Sample preparation	vitrification	
Grid type	UltrAUfoil R1.2/1.3 (300 mesh)	
**EM data collection**		
Microscope	Titan Krios G3i	
Voltage (kV)	300	
Spherical aberration Cs (mm)	2.7	
Condenser C2 aperture size (µm)	70	
Objective aperture size (µm)	100	
Camera	Falcon III	
Pixel size (Å)	0.862	
Total dose (electron*Å^{-2})	31	
Exposure time (sec)	40	
Number of frames	42	
Exposures per hole	2	
Energy filter	None	
Defocus range (µm)	-2.6 to -0.5	
# micrographs collected	1,572	5,260
% micrographs used	82	86
**EM data processing**		
Software	cryoSPARC v4.4	
Picked particles	1,400,859	4,953,014
Particles after 2D classification	133,365	1,194,261
Particles after 3D sorting	120,264	
Resolution (FSC 0.143, Å)	3.6	
**Model building and refinement**		
Software for building	Coot 0.9.8.92	
Residues build	327-736	
Software for refinement	PHENIX 1.21-5207	
Composition (#)<br>Chains<br>Atoms<br>Residues<br>Water<br>Ligands	 3<br>6105 (Hydrogens: 0)<br>Protein: 819<br>0<br>0	
Bonds (RMSD)<br>Length (Å) (# > 4σ)<br>Angles (°) (# > 4σ)	 0.005<br>1.024	
Ramachandran plot (%)<br>Outliers<br>Allowed<br>Favored	 0.86<br>12.70<br>86.44	
Rotamer Outliers (%)	0.0	
MolProbity score	1.99	

Geph^{WT}-GlyR loop	
**Sample conditions**	
Sample concentration	0.005 mg/ml
GlyR-loop	3x fold excess
Sample preparation	Negative stain w/ uranyl formate
Grid type	Continuous carbon (200 mesh)
**EM data collection**	
Microscope	Talos L120C
Voltage (kV)	120
Spherical aberration Cs (mm)	2.7
Condenser C2 aperture size (µm)	70
Objective aperture size (µm)	100
Camera	Ceta16M
Pixel size (Å)	1.86
Total dose (electron*Å^{-2})	40
Exposure time (sec)	1
Number of frames	N/A
Exposures per hole	1
Energy filter	None
Defocus range (µm)	-2.0 to -0.3
# micrographs collected	1,896
% micrographs used	86
**EM data processing**	
Software	cryoSPARC v4.4
Picked particles	504,230
Particles after 2D classification	115,959
Particles after 3D sorting	GephG+E: 27,335 GephE+E: 2,278
Resolution (FSC 0.143, Å)	GephG+E: 6.3 GephE+E: 15.5

Geph^{WT}	
**Sample conditions**	
Sample concentration	0.005 mg/ml
GlyR-loop	none
Sample preparation	Negative stain w/ uranyl formate
Grid type	Continuous carbon (200 mesh)
**EM data collection**	
Microscope	Talos L120C
Voltage (kV)	120
Spherical aberration Cs (mm)	2.7
Condenser C2 aperture size (µm)	70
Objective aperture size (µm)	100
Camera	Ceta16M
Pixel size (Å)	1.86
Total dose (electron*Å^{-2})	40
Exposure time (sec)	1
Number of frames	N/A
Exposures per hole	1
Energy filter	None
Defocus range (µm)	-2.0 to -0.3
# micrographs collected	2,027
% micrographs used	97
**EM data processing**	
Software	cryoSPARC v4.4
Picked particles	650,399
Particles after 2D classification	119,786
Particles after 3D sorting	12,952
Resolution (FSC 0.143, Å)	6.8

Geph^{WT}-GlyR loop	
**Sample conditions**	
Sample concentration	0.5 mg/ml
GlyR-loop	3x fold excess
Sample preparation	vitrification
Grid type	UltrAUfoil R1.2/1.3 (300 mesh)
**EM data collection**	
Microscope	Titan Krios G3i
Voltage (kV)	300
Spherical aberration Cs (mm)	2.7
Condenser C2 aperture size (µm)	70
Objective aperture size (µm)	100
Camera	Falcon III
Pixel size (Å)	0.862
Total dose (electron*Å^{-2})	31
Exposure time (sec)	40
Number of frames	42
Exposures per hole	2
Energy filter	None
Defocus range (µm)	-2.6 to -0.5
# micrographs collected	1,006
% micrographs used	94
**EM data processing**	
Software	cryoSPARC v4.4
Picked particles	486,680
Particles after 2D classification	256,399

Geph^{WT}	
**Sample conditions**	
Sample concentration	0.5 mg/ml
GlyR-loop	none
Sample preparation	vitrification
Grid type	UltrAUfoil R1.2/1.3 (300 mesh)
**EM data collection**	
Microscope	Titan Krios G3i
Voltage (kV)	300
Spherical aberration Cs (mm)	2.7
Condenser C2 aperture size (µm)	70
Objective aperture size (µm)	100
Camera	Falcon III
Pixel size (Å)	0.862
Total dose (electron*Å^{-2})	31
Exposure time (sec)	40
Number of frames	42
Exposures per hole	2
Energy filter	None
Defocus range (µm)	-2.6 to -0.5
# micrographs collected	2468
% micrographs used	83
**EM data processing**	
Software	cryoSPARC v4.4
Picked particles	296,216

Geph$^{\Delta SDII}$- GlyR loop	
**Sample conditions**	
Sample concentration	0.5 mg/ml
GlyR-loop	3x fold excess
Sample preparation	vitrification
Grid type	UltrAUfoil R1.2/1.3 (300 mesh)
**EM data collection**	
Microscope	Titan Krios G3i
Voltage (kV)	300
Spherical aberration Cs (mm)	2.7
Condenser C2 aperture size (µm)	70
Objective aperture size (µm)	100
Camera	Falcon III
Pixel size (Å)	0.862
Total dose (electron*Å^{-2})	31
Exposure time (sec)	40
Number of frames	42
Exposures per hole	2
Energy filter	None
Defocus range (µm)	-2.6 to -0.5
# micrographs collected	2459
% micrographs used	93
**EM data processing**	
Software	cryoSPARC v4.4
Picked particles	854,392
Particles after 2D classification	159,109

Geph^{G375D}- GlyR loop	
**Sample conditions**	
Sample concentration	0.5 mg/ml
GlyR-loop	3x fold excess
Sample preparation	vitrification
Grid type	UltrAUfoil R1.2/1.3 (300 mesh)
**EM data collection**	
Microscope	Titan Krios G3i
Voltage (kV)	300
Spherical aberration Cs (mm)	2.7
Condenser C2 aperture size (µm)	70
Objective aperture size (µm)	100
Camera	Falcon III
Pixel size (Å)	0.862
Total dose (electron*Å^{-2})	31
Exposure time (sec)	40
Number of frames	42
Exposures per hole	2
Energy filter	None
Defocus range (µm)	-2.6 to -0.5
# micrographs collected	1949
% micrographs used	94
**EM data processing**	
Software	cryoSPARC v4.4
Picked particles	2,406,057
Particles after 2D classification	562,918
Particles after 3D sorting	283,888
Resolution (FSC 0.143, Å)	3.4

Geph^{D422N}- GlyR loop	
**Sample conditions**	
Sample concentration	0.5 mg/ml
GlyR-loop	3x fold excess
Sample preparation	vitrification
Grid type	UltrAUfoil R1.2/1.3 (300 mesh)
**EM data collection**	
Microscope	Titan Krios G3i
Voltage (kV)	300
Spherical aberration Cs (mm)	2.7
Condenser C2 aperture size (µm)	70
Objective aperture size (µm)	100
Camera	Falcon III
Pixel size (Å)	0.862
Total dose (electron*Å^{-2})	31
Exposure time (sec)	40
Number of frames	42
Exposures per hole	2
Energy filter	None
Defocus range (µm)	-2.6 to -0.5
# micrographs collected	2609
% micrographs used	87
**EM data processing**	
Software	cryoSPARC v4.4
Picked particles	2,259,602
Particles after 2D classification	256,399
Particles after 3D sorting	78,377
Resolution (FSC 0.143, Å)	3.1

Geph^{R379D}- GlyR loop	
**Sample conditions**	
Sample concentration	0.5 mg/ml
GlyR-loop	3x fold excess
Sample preparation	vitrification
Grid type	UltrAUfoil R1.2/1.3 (300 mesh)
**EM data collection**	
Microscope	Titan Krios G3i
Voltage (kV)	300
Spherical aberration Cs (mm)	2.7
Condenser C2 aperture size (µm)	70
Objective aperture size (µm)	100
Camera	Falcon III
Pixel size (Å)	0.862
Total dose (electron*Å^{-2})	31
Exposure time (sec)	40
Number of frames	42
Exposures per hole	2
Energy filter	None
Defocus range (µm)	-2.6 to -0.5
# micrographs collected	2,102
% micrographs used	70
**EM data processing**	
Software	cryoSPARC v4.4
Picked particles	2,259,602
Particles after 2D classification	1,434,120
Particles after 3D sorting	18,615
Resolution (FSC 0.143, Å)	3.5
