# Supplementary material for: Gephyrin filaments represent the molecular basis of inhibitory postsynaptic densities
Source: Nat Commun. 2025 Sep 16;16:8293. doi: 10.1038/s41467-025-63748-w (PMC12441120; doi:10.1038/s41467-025-63748-w)
Supplement: Supplementary file 2 — Reporting Summary [file 41467_2025_63748_MOESM2_ESM.pdf]

nature portfolio

Corresponding author(s): Günter Schwarz, Elmar Behrmann

Last updated by author(s): Aug 19, 2025

Reporting Summary

Nature Portfolio wishes to improve the reproducibility of the work that we publish. This form provides structure for consistency and transparency in reporting. For further information on Nature Portfolio policies, see our Editorial Policies and the Editorial Policy Checklist.

Statistics

For all statistical analyses, confirm that the following items are present in the figure legend, table legend, main text, or Methods section.

n/a	Confirmed	
☐	☒	The exact sample size (*n*) for each experimental group/condition, given as a discrete number and unit of measurement
☐	☒	A statement on whether measurements were taken from distinct samples or whether the same sample was measured repeatedly
☐	☒	The statistical test(s) used AND whether they are one- or two-sided<br>*Only common tests should be described solely by name; describe more complex techniques in the Methods section.*
☒	☐	A description of all covariates tested
☐	☒	A description of any assumptions or corrections, such as tests of normality and adjustment for multiple comparisons
☐	☒	A full description of the statistical parameters including central tendency (e.g. means) or other basic estimates (e.g. regression coefficient) AND variation (e.g. standard deviation) or associated estimates of uncertainty (e.g. confidence intervals)
☐	☒	For null hypothesis testing, the test statistic (e.g. *F*, *t*, *r*) with confidence intervals, effect sizes, degrees of freedom and *P* value noted<br>*Give P values as exact values whenever suitable.*
☒	☐	For Bayesian analysis, information on the choice of priors and Markov chain Monte Carlo settings
☒	☐	For hierarchical and complex designs, identification of the appropriate level for tests and full reporting of outcomes
☒	☐	Estimates of effect sizes (e.g. Cohen's *d*, Pearson's *r*), indicating how they were calculated

Our web collection on statistics for biologists contains articles on many of the points above.

Software and code

Policy information about availability of computer code

Data collection	EPU (version 2.12), LAS X (Leica)
Data analysis	cryoSPARC (version 4.4), ChimeraX (version 1.9.dev202410120941), Phenix (version 1.21-5207), Coot (version 0.9.8.92), Origin 7, ImageJ/FIJI (version 1.53c)

For manuscripts utilizing custom algorithms or software that are central to the research but not yet described in published literature, software must be made available to editors and reviewers. We strongly encourage code deposition in a community repository (e.g. GitHub). See the Nature Portfolio guidelines for submitting code & software for further information.

Data

Policy information about availability of data

All manuscripts must include a data availability statement. This statement should provide the following information, where applicable:

- Accession codes, unique identifiers, or web links for publicly available datasets
- A description of any restrictions on data availability
- For clinical datasets or third party data, please ensure that the statement adheres to our policy

Unless otherwise stated, all data supporting the results of this study can be found in the article, supplementary, and source data files.
Plasmids have been made available via Addgene [https://www.addgene.org/]. The EM maps for GephEWT, GephEG375D, GephED422N, and GephER379D have been deposited in the EMDB under accession codes EMD-51644 [https://www.ebi.ac.uk/emdb/EMD-51644], EMD-54820 [https://www.ebi.ac.uk/emdb/

EMD-54820], EMD-54821 [https://www.ebi.ac.uk/emdb/EMD-54821], EMD-54824 [https://www.ebi.ac.uk/emdb/EMD-54824]. The raw electron microscopy imaging data are available on request due to their large size. Atomic coordinates for GephEWT have been deposited in the Protein Data Bank under the accession code 9GW9. Source data are provided with this paper.

Research involving human participants, their data, or biological material

Policy information about studies with human participants or human data. See also policy information about sex, gender (identity/presentation), and sexual orientation and race, ethnicity and racism.

Reporting on sex and gender	Our research did not involve human participants, their data or biological material. Sex and gender were not considered in the study design.
Reporting on race, ethnicity, or other socially relevant groupings	Our research did not involve human participants, their data or biological material. Race, ethnicity or other social groupings were not considered in the study design.
Population characteristics	see above
Recruitment	Our research did not involve human participants, their data or biological material.
Ethics oversight	see above

Note that full information on the approval of the study protocol must also be provided in the manuscript.

Field-specific reporting

Please select the one below that is the best fit for your research. If you are not sure, read the appropriate sections before making your selection.

☒ Life sciences ☐ Behavioural & social sciences ☐ Ecological, evolutionary & environmental sciences

For a reference copy of the document with all sections, see nature.com/documents/nr-reporting-summary-flat.pdf

Life sciences study design

All studies must disclose on these points even when the disclosure is negative.

Sample size	For cryoEM: An adequate number of micrographs were recorded to ensure high resolultion reconstruction or at least 2D classification of particles.<br>For biochemistry studies: 3 technical replicates from one purification<br>For cell culture studies: Per condition 20 regions were imaged from four independent primary neuronal cultures.
Data exclusions	In general, no data was excluded from analysis. For the cryo-EM reconstruction, micrographs with poor quality scores (drift, CTFfit, astigmatism) were excluded from further analysis. This was done before evaluating individual particle images from the micrographs and thus in a blinded fashion.
Replication	In general for EM experiments data from one EM-grid for processing was considered. In case of GephE-WT two datasets (tilted and untilted) from two technical replicas were combined and processed. For sedimentation experiments three technical replicas were considered. For ITC experiments a representative result of a total vour experiments is shown. For cell culture experiments 20 biological replicas were used in total per gephyrin variant.
Randomization	Randomization is not relevant for EM, SEC, ITC, cell culture and SDS-PAGE experiments shown in this study as they are biological studies with well-defined parameters and statistics and their results show low variations that are numerically defined.
Blinding	In general, validation of all experiments presented in this study relied completely on statistics and well defined parameters thus blinded evaluation of data was not necessary. A bias due to omission of blinding is not expected to affect the experiments performed in this study.

Reporting for specific materials, systems and methods

We require information from authors about some types of materials, experimental systems and methods used in many studies. Here, indicate whether each material, system or method listed is relevant to your study. If you are not sure if a list item applies to your research, read the appropriate section before selecting a response.

Materials & experimental systems

n/a	Involved in the study
☐	☒ Antibodies
☐	☒ Eukaryotic cell lines
☒	☐ Palaeontology and archaeology
☐	☒ Animals and other organisms
☒	☐ Clinical data
☒	☐ Dual use research of concern
☒	☐ Plants

Methods

n/a	Involved in the study
☒	☐ ChIP-seq
☒	☐ Flow cytometry
☒	☐ MRI-based neuroimaging

Antibodies

Antibodies used	primary:<br>anti-vesicular GABA transporter (vGAT) (1:1000, #131003) for inhibitory presynaptic terminals, anti-GABAAR γ2 (1:500, #224004) for postsynaptic GABAARs<br>secondary<br>goat anti-rabbit AlexaFluor 488 (1:500, #A-11034, Thermo Fisher Scientific) and goat anti-guinea pig AlexaFluor 647 (1:500, #ab150187, Abcam)
Validation	KO validation, see company website https://sysy.com/product/131003<br>anti-vesicular GABA transporter (vGAT) (#131003) for inhibitory presynaptic terminals<br><br>validation for various applications, see company website https://sysy.com/product/224004<br>anti-GABAAR γ2 (1:500, #224004) for postsynaptic GABAARs

Eukaryotic cell lines

Policy information about cell lines and Sex and Gender in Research

Cell line source(s)	HEK293T cells (DSMZ no. ACC 635) for AAV production
Authentication	cell line was not authenticated
Mycoplasma contamination	cell line tested negative for mycoplasma contamination
Commonly misidentified lines (See ICLAC register)	n/a

Animals and other research organisms

Policy information about studies involving animals; ARRIVE guidelines recommended for reporting animal research, and Sex and Gender in Research

Laboratory animals	Gphn flox/flox; C57BL/6NRj
Wild animals	n/a
Reporting on sex	Dissociated primary hippocampal cultures were prepared from embryos of either sex.
Field-collected samples	n/a
Ethics oversight	We complied with all relevant ethical regulations for animal testing and research. Experiments were approved by the local research ethics committees (Germany, Landesamt für Natur, Umwelt und Verbraucherschutz Nordrhein-Westfalen, reference and 2021.A450).

Note that full information on the approval of the study protocol must also be provided in the manuscript.

Plants

Seed stocks	*Report on the source of all seed stocks or other plant material used. If applicable, state the seed stock centre and catalogue number. If plant specimens were collected from the field, describe the collection location, date and sampling procedures.*
Novel plant genotypes	*Describe the methods by which all novel plant genotypes were produced. This includes those generated by transgenic approaches, gene editing, chemical/radiation-based mutagenesis and hybridization. For transgenic lines, describe the transformation method, the number of independent lines analyzed and the generation upon which experiments were performed. For gene-edited lines, describe the editor used, the endogenous sequence targeted for editing, the targeting guide RNA sequence (if applicable) and how the editor was applied.*
Authentication	*Describe any authentication procedures for each seed stock used or novel genotype generated. Describe any experiments used to assess the effect of a mutation and, where applicable, how potential secondary effects (e.g. second site T-DNA insertions, mosiacism, off-target gene editing) were examined.*
