# Supplementary figures and images for: Gephyrin filaments represent the molecular basis of inhibitory postsynaptic densities
Source: Nat Commun. 2025 Sep 16;16:8293. doi: 10.1038/s41467-025-63748-w (PMC12441120; doi:10.1038/s41467-025-63748-w)

## Slide 1
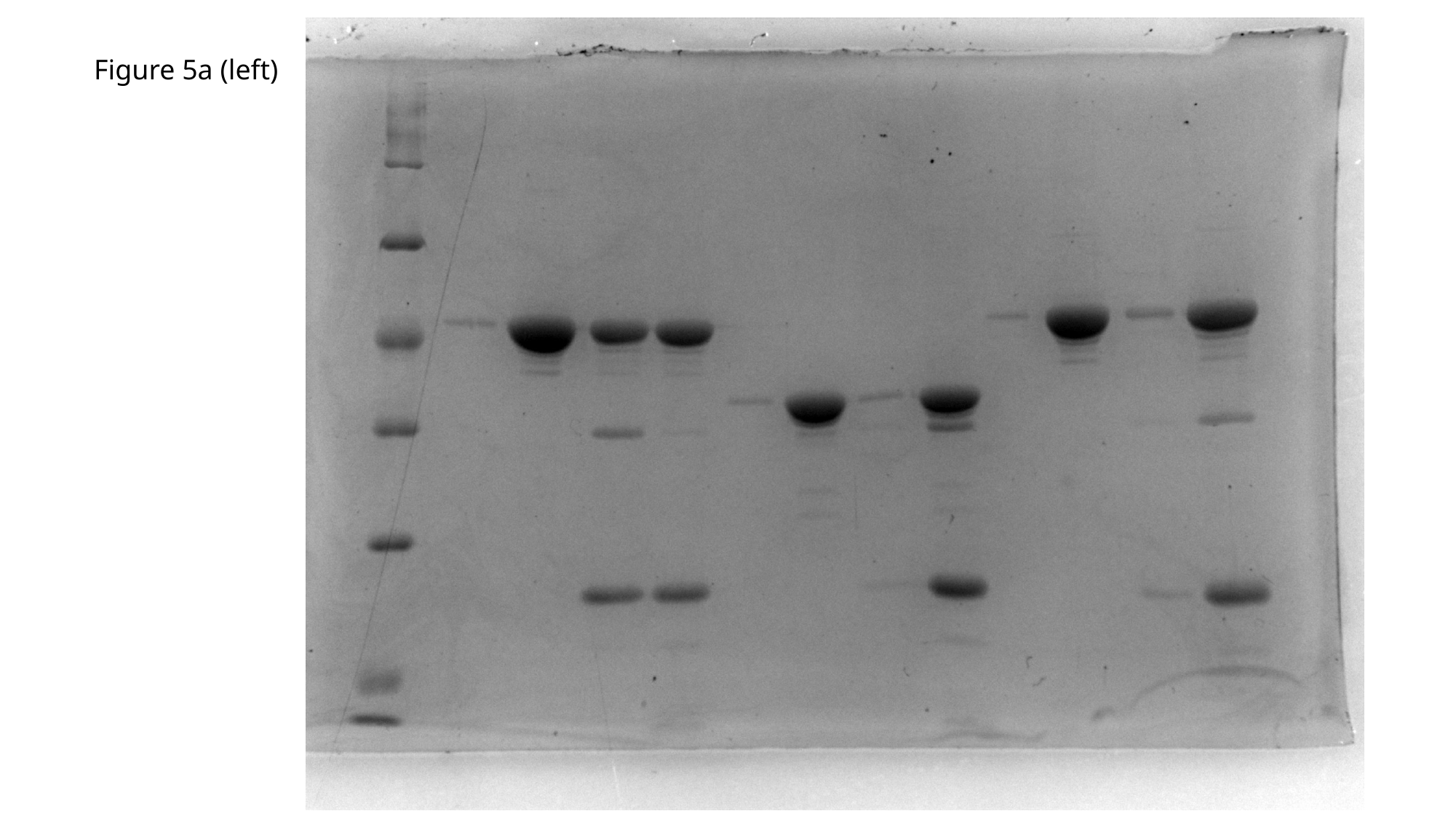

Figure 5a (left)

## Slide 2
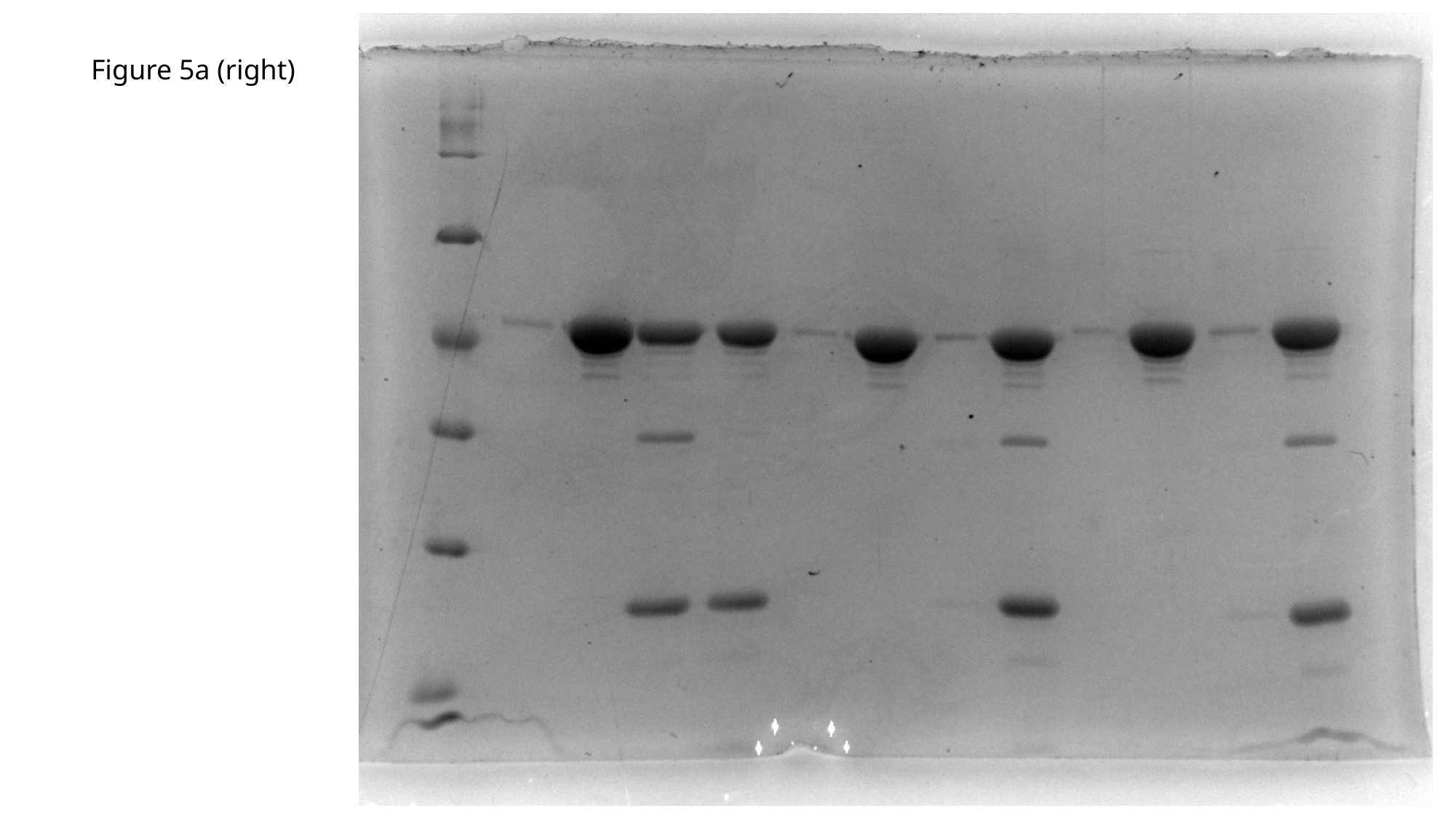

Figure 5a (right)

## Slide 3
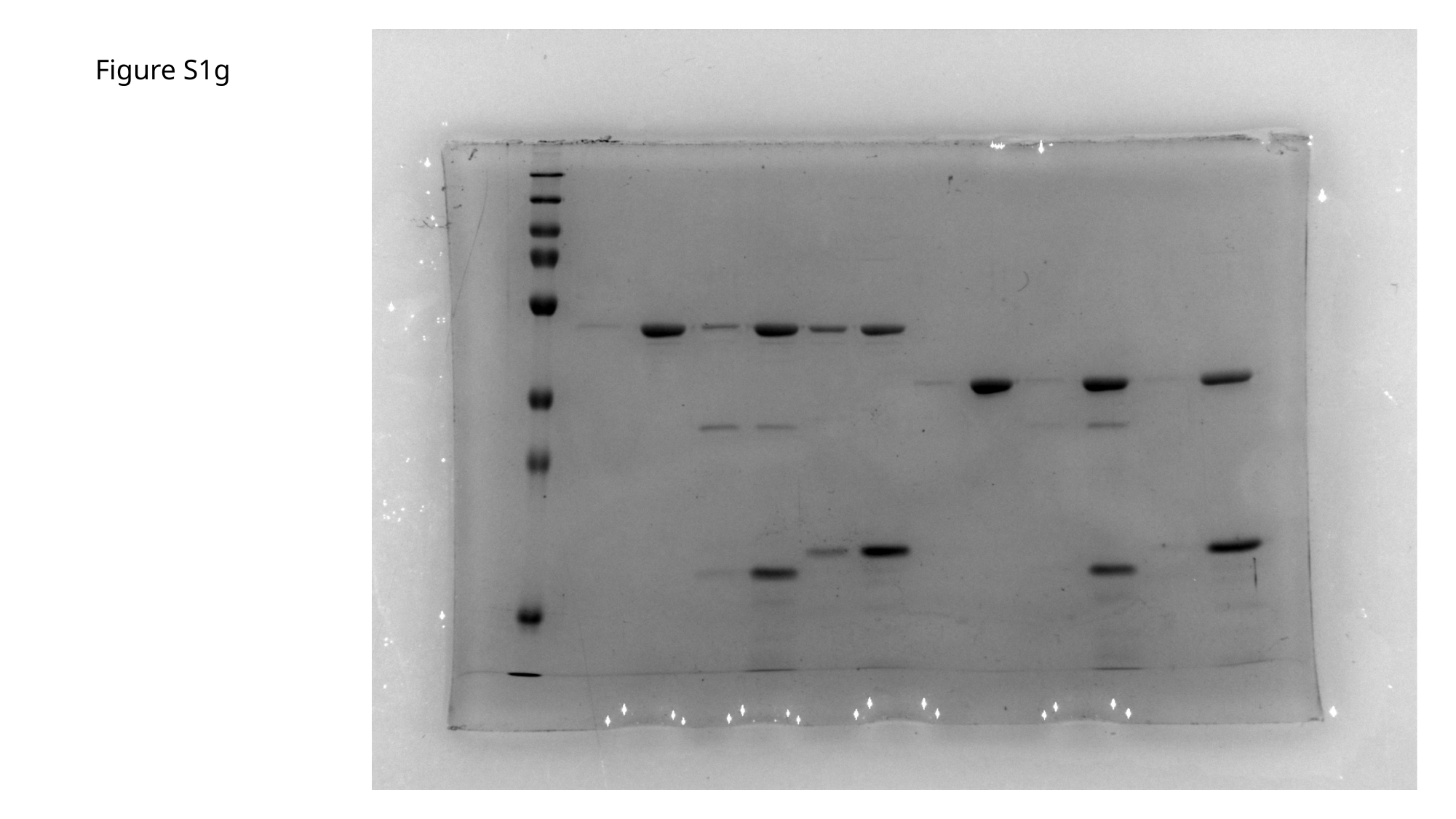

Figure S1g

Supplement: Supplementary file 4 — Source data [file 41467_2025_63748_MOESM4_ESM.xlsx › Uncropped Images Fig 5a S1g.pptx]
